# Purification and Characterization of a Thermo- and pH-Stable Laccase From the Litter-Decomposing Fungus *Gymnopus luxurians* and Laccase Mediator Systems for Dye Decolorization

**DOI:** 10.3389/fmicb.2021.672620

**Published:** 2021-08-03

**Authors:** Yue Sun, Zi-Lu Liu, Bo-Yang Hu, Qing-Jun Chen, Ai-Zhen Yang, Qiu-Ying Wang, Xiao-Feng Li, Jia-Yan Zhang, Guo-Qing Zhang, Yong-Chang Zhao

**Affiliations:** ^1^Beijing Key Laboratory for Agricultural Application and New Technique, Beijing Advanced Innovation Center for Tree Breeding by Molecular Design, College of Plant Science and Technology, Beijing University of Agriculture, Beijing, China; ^2^Key Laboratory for Northern Urban Agriculture of Ministry of Agriculture and Rural Affairs, College of Bioscience and Resources Environment, Beijing University of Agriculture, Beijing, China; ^3^College of Biological Sciences and Technology, Beijing Forestry University, Beijing, China; ^4^Institute of Biotechnology and Germplasmic Resource, Yunnan Academy of Agricultural Sciences, Kunming, China

**Keywords:** *Gymnopus luxurians*, laccase, laccase mediator system, dye decolorization, purification

## Abstract

An extracellular laccase (GLL) was purified from fermentation broth of the litter-decomposing fungus *Gymnopus luxurians* by four chromatography steps, which resulted in a high specific activity of 118.82 U/mg, purification fold of 41.22, and recovery rate of 42.05%. It is a monomeric protein with a molecular weight of 64 kDa and N-terminal amino acid sequence of AIGPV TDLHI, suggesting that GLL is a typical fungal laccase. GLL demonstrated an optimum temperature range of 55°C–65°C and an optimum pH 2.2 toward 2,2′-azino-bis(3-ethylbenzothiazoline-6-sulfonic acid) (ABTS). It displayed considerably high thermostability and pH stability with about 63% activity retained after 24 h at 50°C, and 86% activity retained after 24 h at pH 2.2, respectively. GLL was significantly enhanced in the presence of K^+^, Na^+^, and Mg^2+^ ions. It demonstrated *K*_*m*_ of 539 μM and *k*_*cat*_/*K*_*m*_ of 140 mM^–1^⋅s^–1^ toward ABTS at pH 2.2 and 37°C. Acetosyringone (AS) and syringaldehyde (SA) were the optimal mediators of GLL (0.4 U/ml) for dye decolorization with decolorization rates of about 60%–90% toward 11 of the 14 synthetic dyes. The optimum reaction conditions were determined to be mediator concentration of 0.1 mM, temperature range of 25°C –60°C, and pH 4.0. The purified laccase was the first laccase isolated from genus *Gymnopus* with high thermostability, pH stability, and effective decolorization toward dyes, suggesting that it has potentials for textile and environmental applications.

## Introduction

Laccases (EC 1.10.3.2, p-diphenol: dioxygen oxidoreductases) are copper-containing polyphenol oxidases. They have been studied for more than one century due to their high oxidation ability toward a wide range of aromatic and non-aromatic compounds (Rivera-Hoyos et al., 2013; Arregui et al., 2019). Although laccases are widely distributed in plants, insects, and microorganisms, fungal laccases are more efficient and widely reported (Baldrian, 2006; Rivera-Hoyos et al., 2013). Due to the high catalytic efficiency and broad substrate specificity, fungal laccases are of great potential in various industries of delignification, biopulping, biobleaching, wastewater treatment, and detoxification of pollutants (Baldrian, 2006; Canas and Camarero, 2010; Dwivedi et al., 2011; Arregui et al., 2019).

The stability and efficiency are the most important factors that restrict the application of fungal laccases. Most fungal laccases are functional at an optimal temperature range of 40°C –70°C and an acidic optimal pH range of 2.0–6.0 (Baldrian, 2006; Viswanath et al., 2014). However, many of them are easily inactivated at high temperature or low pH. Thermostable or pH stable laccases possess higher application values under industrial conditions. Meanwhile, laccases possess relatively low redox potential (≤ 0.8 V) and would be restricted to the oxidation of many substrates with higher redox potential (≥ 1.3 V) (Canas and Camarero, 2010). Thankfully, this limitation has been overcome by using several small-molecule redox mediators in the so-called laccase mediator systems (LMS). These redox mediators, such as 2,2′-azino-bis (3-ethylbenzothiazoline-6-sulfonic acid) (ABTS) and 1-hydroxybenzotriazole (HBT), can act as electron shuttles during the oxidation process and possess higher redox potential or active sites (Morozova et al., 2007; Canas and Camarero, 2010). Up till now, mediators and LMS were widely investigated and applied in various fields especially in degradation and detoxification of pollutants (Canas and Camarero, 2010; Daassi et al., 2012; Apriceno et al., 2019).

At present, more than 100 fungal laccases have been purified and characterized, most of which were isolated from white rotting fungi (Rivera-Hoyos et al., 2013; Zhang et al., 2018). Meanwhile, there are a large number of litter-decomposing fungi in the forest surface, which play an important role in the process of forest litter biodegradation. However, few literatures on laccases from litter-decomposing fungi have been reported. It might be due to the fact that fruiting bodies of some species are relatively small and difficult to isolate, while others are difficult to cultivate artificially. *Gymnopus* is a large genus of litter-decomposing fungi in the family Marasmiaceae distributed worldwide and contains more than 300 species (Antonin et al., 2013). Low laccase activity of *Gymnopus sapineus* can be detected in the Pb-containing soil (Kahkonen et al., 2008). *Gymnopus erythropus* demonstrated a low laccase activity of 4.2 mU/ml after a 35-day liquid fermentation (Vetrovsky et al., 2013). Up to now, the genomes of *Gymnopus androsaceus*, *Gymnopus earleae*, and *Gymnopus luxurians* have been sequenced, several hypothetical laccase genes have been reported, while no laccase protein from genus *Gymnopus* was obtained (Grigoriev et al., 2014; Barbi et al., 2020).

In our pervious study, a pure culture strain YAASM5159 of *G. luxurians* was isolated from fruiting bodies on the forest floor. It can be artificially cultivated, and fruiting bodies were edible. Moreover, the strain possessed considerably high extracellular laccase activity during liquid fermentation (Sun et al., 2021). In order to expand the research of laccases from litter-decomposing fungi, we focused on the laccase purification, enzymatic characteristics, and application on dye decolorization of *G. luxurians* strain YAASM5159.

## Materials and Methods

### Chemical Agents and Dyes

ABTS, acrylamide, bisacrylamide, glycine, sodium dodecyl sulfate (SDS), and Tris-base were purchased from Sigma-Aldrich Chem. Co. (United States). DEAE-Sepharose, Q-Sepharose, SP-Sepharose, Superdex 75 HR 10/30 column, and molecular mass standards for FPLC were obtained from GE Healthcare Co. Ltd (Boston, MA, United States). The protein marker for SDS-PAGE was purchased from Biomed SciTech Co., Ltd. (Beijing, China). Synthetic dyes including active red (AR), bromophenol blue (BB), Coomassie brilliant blue G250 (CBB), Evans blue (EB), eriochrome black T (EBT), malachite green (MG), methyl orange (MO), reactive black (RB), reactive brilliant blue (RBB), reactive orange (RO), reactive red (RR), reactive turquoise blue (RTB), reactive yellow (RY), trypan blue (TB), and mediators including acetosyringone (AS), HBT, syringaldehyde (SA), 2,2,6,6-tetramethylpiperidine-1-oxyl (TEMPO), vanillin (VAL), and violuric acid (VIA) were purchased from Sinopharm Chemical Reagent Co., Ltd. (Shanghai, China). Other chemicals used were of analytical reagent grade.

### Strain and Culture Condition

*Gymnopus luxurians* strain YAASM5159 was previously collected from Chuxiong of Yunnan Province in China. The strain was maintained through monthly transfer on potato dextrose agar (PDA) medium at 4°C. The PDA medium contained the following (g/L of distilled water): potato 200, glucose 20, agar 20, and pH range of 7.0–7.2. The newly activated strain was inoculated on PDA plates (90 mm) and grown at 28°C. When the mycelia overgrew on the plate, the full-plate colony with the medium was moved into a sterile homogenizer and homogenized with 100 ml sterile potato dextrose broth (PDB) medium. The obtained homogenate was inoculated into a 500-ml Erlenmeyer flask containing 150 ml PDB medium at an inoculation amount of 10% (V/V). The cultivation was carried out in a dark chamber under 150 rpm shaking speed at 28°C for 6 days. The fermentation broth was subsequently used as laccase primary extract.

### Laccase Activity Assay

Laccase activity was spectrophotometrically determined by using ABTS as the substrate and measuring the increase in absorbance at 420 nm (ε_420_ = 3.6 × 10^4^ M^–1^ cm^–1^) (Si et al., 2013). Laccase solution (5 μl) was incubated with 0.6 mM ABTS solution (195 μl, in 50 mM sodium acetate buffer, pH 4.0) at 37°C in water bath for 5 min, followed by an addition of 10% TCA (200 μl) to end the reaction. One unit of enzyme activity (U) was defined as the amount of enzyme required to oxidize 1 μmol ABTS per min (Othman et al., 2018).

### Purification of Laccase

After 6-day cultivation, the fermented broth was centrifuged at 4°C and 6,000 rpm for 30 min, followed by dialysis in distilled water overnight. Subsequently, the crude enzyme solution was separated on anion exchange chromatography of DEAE-Sepharose previously equilibrated with 10 mM Tris–HCl buffer (pH 7.5). After the unadsorbed proteins had been eluted, the adsorbed proteins were successively eluted with 50, 150, and 800 mM NaCl in the same buffer. All obtained fractions were monitored for laccase activity. A laccase-rich fraction was subsequently pooled and dialyzed for further purification on cation exchange chromatography of SP Sepharose (10 mM HAc-NaAc, pH 4.0). After sampling, the solution was eluted successively with 0, 50, and 1000 mM NaCl in the same buffer. The laccase active fraction was then subjected to anion exchange chromatography Q-Sepharose (10 mM Tris–HCl buffer, pH 7.2). After removal of unadsorbed proteins, adsorbed proteins were eluted with a linear concentration gradient of NaCl (0–1,000 mM) in the same buffer. Ultimately, the laccase active fraction was purified by fast protein liquid chromatography (FPLC) on a Superdex 75 HR 10/30 column (0.15 M NH_4_HCO_3_ buffer, pH 8.5) using an AKTA Purifier (GE Healthcare, United States).

### N-Terminal and Inner Amino Acid Sequencing

The molecular weight (MW) of the purified laccase (abbreviated as GLL) was determined by both FPLC and sodium dodecyl sulfate-polyacrylamide gel electrophoresis (SDS-PAGE). Molecular weight of the native protein was determined based on a standard curve of Log MW vs. elution volume of molecular mass standards for FPLC. SDS-PAGE was performed using the standard procedure with a 12% resolving gel and a 5% stacking gel. The MW of denatured proteins was determined by the standard curve of Log MW vs. relative mobilities of molecular mass standards for SDS-PAGE (Wang et al., 2017). After SDS-PAGE, the purified laccase band was further analyzed for N-terminal and inner amino acid sequencing. N-Terminal amino acid sequencing was performed by using an HP G1000A Edman degradation unit (Hewlett-Packard Company, Palo Alto, CA, United States) and an HP 1000 HPLC system (Hewlett-Packard Company, United States). The inner amino acid sequencing was analyzed by nano-liquid chromatography coupled with linear trap Quadrupole Orbitrap mass spectrometry (LC-LTQ-Orbitrap-MS, Thermo Fisher, Waltham, MA, United States). The data were acquired using Xcalibur software (Thermo Electron, United States). Peptide fingerprints were identified by homology against the microbial laccase database of the National Center for Biotechnology Information (NCBI) (Wang et al., 2018; Yang et al., 2020).

### Enzymatic Characteristics

To determine the optimal temperature of the purified laccase GLL, the reaction mixture of the standard laccase assay above was examined from 25 to 85°C. To determine the optimal pH, laccase activity was tested by using a series of ABTS solution in the pH range of 1.0–2.2 (KCl–HCl buffers, 50 mM) and 2.2–7.2 (citric acid–Na_2_HPO_4_ buffers, 50 mM) instead of the standard ABTS solution at pH 4.0. In the thermostability assay, the purified laccase was pre-incubated at 30°C, 40°C, 50°C, and 60°C for 1, 2, 4, and 24 h, respectively, followed by residual activity determination using the standard laccase assay above. In the pH stability assay, a series of citric acid–Na_2_HPO_4_ buffers (50 mM, pH 2.2, 3.0, 3.8, and 4.2) were used. The purified laccase solutions were co-incubated with assay buffers (1:1, V/V) at 4°C for 1, 2, 4, and 24 h, respectively. The residual activity was immediately measured using the standard laccase assay above. The effects of metal ions and EDTA on the purified laccase were investigated using KCl, NaCl, CaCl_2_, CdCl_2_, CoCl_2_, CuCl_2_, MgCl_2_, MnCl_2_, and EDTA solution (in sodium acetate buffer, pH 4.0, 50 mM) with the final assay concentrations of 1.25, 2.5, 5.0, and 10 mM, respectively. The purified laccase was pre-incubated with the assay reagents (1:1, v/v) at 4°C for 1 h, followed by the standard laccase assay for the residual activity. The purified laccase treated with distilled water was used as the control. Kinetic studies of the purified laccase were carried out using ABTS as substrate in a series concentration ranging from 12.5 to 800 μM at pH 2.2 (citric acid–Na_2_HPO_4_ buffer, 50 mM) and 37°C. The values of *K*_*m*_, *V*_*max*_, *k*_*cat*_, and *k*_*cat*_/*K*_*m*_ were calculated from a Lineweaver–Burk plot (Jeon and Lim, 2017; Wang et al., 2018).

### Optimal Mediators and Reaction Conditions for Dye Decolorization

Dye decolorization capacity of the purified laccase GLL was estimated using 14 kinds of dyes of three types which were investigated including five kinds of azo dyes (EB, EBT, MO, RB, and TB), six kinds of reactive dyes (AR, RBB, RO, RR, RTB, and RY), and three kinds of triphenylmethane dyes (BB, CCB, and MG). To improve the decolorization efficiency of the purified laccase GLL, seven kinds of mediators were used including ABTS, AS, HBT, SA, TEMPO, VAL, and VIA. The reaction mixture containing 10 μl purified laccase (0.4 U/ml) and 190 μl assayed dyes (50 mg/l in pH 4.0 citric acid- Na_2_HPO_4_ buffer, 50 mM) with or without each assayed mediator (0.1 mM) was incubated in a 96-well plate at room temperature and dark condition for 1, 4, 12, and 24 h, respectively. The decolorizing abilities were determined by a microplate reader (Bio-Rad, Hercules, CA, United States) at the maximum absorption wavelength of each assayed dye (listed in Table 1). The decolorization rate was expressed in terms of percentage and calculated as follows: Decolorization rate (%) = 100% × (Initial absorbance - Final absorbance)/Initial absorbance (Si et al., 2013; Wang et al., 2018).

**TABLE 1:** Optical absorption characteristics of 14 kinds of synthetic dyes.

Chemical class	Dyes	λ_*max*_ (nm)
		
Azo dyes	Eriochrome black T (EBT)	540
	Evans blue (EB)	610
	Methyl orange (MO)	460
	Reactive black (RB)	585
	Trypan blue (TB)	600
Reactive dyes	Active red (AR)	440
	Reactive brilliant blue (RBB)	605
	Reactive orange (RO)	441
	Reactive red (RR)	546
	Reactive turquoise blue (RTB)	259
	Reactive yellow (RY)	327
Triphenylmethane dyes	Bromophenol blue (BB)	590
	Coomassie brilliant blue G250 (CBB)	595
	Malachite green (MG)	614

In the optimal reaction condition assay of LMS, two optimal mediators (AS and SA) and four dyes (RB, CBB, MG, and TB) were evaluated. In the optimal mediator concentration assay, 0, 0.05, 0.1, 0.5, and 1.0 mM each mediator were used in the standard LMS decolorization assay above. In the optimal temperature and pH assay, temperatures of 25°C, 37°C, 50°C, and 60°C and pH values of 2.2, 3.0, 4.0, and 5.0 were performed, respectively, in the standard LMS decolorization assay above. After a 4-h incubation, the decolorization rate was determined. Ultimately, decolorization activities of GLL-AS and GLL-SA toward the four dyes were evaluated at mediator concentrations of 0.1 mM, pH 4.0, and 60°C for 1, 2, and 4 h. Subsequently, full-wavelength scanning (300–800 nm) of reaction products was performed using a UV-Vis spectrophotometer (Shanghai Puyuan, 19000 PC, Shanghai, China). In each assay, free dye without any laccase or mediator was used as the blank control, and dye with the laccase was used as the positive control.

### Statistical Analysis

All data were presented as mean ± standard deviation (SD) for three replications for each sample. Statistical analysis was carried out using SPSS (v.25.0) software. The letters denoted the statistical significance according to the Tukey test at *P* < 0.05.

## Results and Discussion

### Laccase Purification and Molecular Weight Determination

**FIGURE 1:**
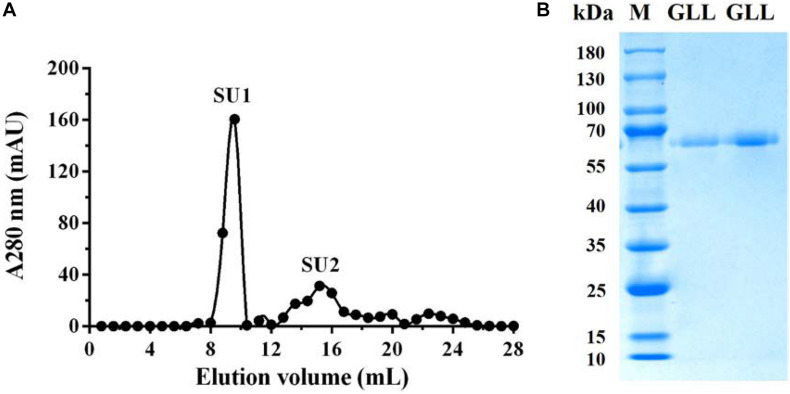
**(A)** FPLC-gel filtration on Superdex 75 HR 10/30 column. Eluent: 0.15 M NH_4_HCO_3_ buffer (pH 8.5). Fraction size: 0.8 ml. Flow rate: 0.8 ml/min. Fraction SU1 represents purified laccase. **(B)** SDS-PAGE of Fraction SU1 from FPLC. GLL, *G. luxurians* laccase, M, Molecular weight markers.

The purified extracellular laccase GLL was obtained following an isolation protocol of three ion-exchange and one-gel-filtration chromatography steps. The purification details are summarized in Table 2. The final chromatography on FPLC yielded the purified laccase in a large fraction SU1 with the native MW of 64 kDa (Figure 1A). Subsequently, GLL appeared as a single band of 64 kDa in SDS-PAGE (Figure 1B). It suggested that the native laccase is a monomeric protein. The purified laccase exhibited an activity of 118.82 U/mg toward ABTS at the standard assay condition with an activity recovery of 42.05% and an overall 41.22-fold purification (Table 2).

**TABLE 2:** Summary of purification procedure of the purified laccase GLL (from 1 l fermentation broth).

Purification step	Yield (mg)	Total activity (U)	Specific activity (U/mg)	Recovery of activity (%)	Purification fold
Fermentation broth	2,083.00	6,004.44	2.88	100.00	1.00
DEAE-cellulose (D3)	208.50	5,364.44	25.73	89.34	8.93
SP-Sepharose (SP1)	61.13	3,803.33	62.22	63.34	21.59
Q-Sepharose (Q1)	30.33	2,980.44	98.26	49.64	34.09
FPLC (SU1)	21.25	2,525.00	118.82	42.05	41.22

Although many fungal laccases were purified and reviewed (Baldrian, 2006; Rivera-Hoyos et al., 2013; Arregui et al., 2019), few laccases were reported from litter-decomposing fungi. Up till now, no laccase protein purified from *Gymnopus* genus has been reported. The present laccase GLL from *G. luxurians* strain YAASM5159 is a monomeric protein with MW of 64 kDa, suggesting that it was similar with typical fungal laccases in both molecular weight (about 50–90 kDa) and molecular composition (monomeric) (Baldrian, 2006; Rivera-Hoyos et al., 2013). A characteristic comparison of the present laccase GLL and other *Marasmius* laccases also from family Marasmiaceae is summarized in Table 3. All the compared Marasmiaceae laccases are monomeric with MW of 53–75 kDa, while specific activity, recovery of activity, and purification fold of the laccases are rather changeable. Laccase from *M. scorodonius* demonstrated the highest specific activity of 432.8 U/mg, but lowest recovery rate of 5% (Jeon and Lim, 2017). Another laccase from *Marasmius* sp. BBKAV79 was purified with the lowest specific activity of 0.226 U/mg and a final yield of 13.5% (Vantamuri and Kaliwal, 2016). It suggested that laccases from family Marasmiaceae showed high diversity in MW and specific activity.

**TABLE 3:** Characteristic comparison of GLL from *G. luxurians* strain YAASM5159 (this study) and other laccases from family Marasmiaceae.

	*G. luxurians* (this study)	*Marasmius* sp. (Schuckel et al., 2011)	*Marasmius* sp. BBKAV79 (Vantamuri and Kaliwal, 2016)	*M. quercophilus* C7 (Farnet et al., 2002)	*M. quercophilus* C19 (Farnet et al., 2004)	*M. scorodonius* (Jeon and Lim, 2017)
Molecular weight (kDa)	64	53	75	65	60	67
Molecular composition	Monomeric	Monomeric	Monomeric	Monomeric	Monomeric	Monomeric
Specific activity (U/mg)	118.82	51.2	0.226	n.d.	n.d.	432.8
Recovery of activity (%)	42.05	60	13.5	n.d.	n.d.	5
Purification fold	41.22	171	3.77	n.d.	n.d.	206
Optimal pH (toward ABTS)	2.2	3.0	n.d.	2.6	4.0	3.4
Optimal temperature (toward ABTS)	55°C–65°C	45°C–55°C	n.d.	80°C	80°C	75°C
*K*_*m*_ value (μM)	539	3.9	3030	7700	113	27
*k*_*cat*_/*K*_*m*_ (mM^–1^⋅s^–1^)	140	30,769	n.d.	n.d.	n.d.	2609

*n.d., not determined.*

### N-Terminal and Inner Amino Acid Sequencing

The N-terminal amino acid sequence of GLL was determined to be AIGPV TDLHI which shared 100% sequence homology with that of *Panus rudis* (AAR13230.1) and *Lentinula edodes* (AET86511.1), but 80% sequence homology with that of *M. scorodonius* and *Marasmius* sp. (AIGPV ADLVI) (Schuckel et al., 2011; Jeon and Lim, 2017). Up to now, the genomes of *G. androsaceus*^1^, *G. earleae*^2^, and *G. luxurians*^3^ have been sequenced. The N-terminal amino acid sequence of GLL possessed 70%–90% similarity with that of hypothetical laccases from *G. androsaceus* (AIGPV TDLNI and SIGPV ADLNI) and *G. earleae* (AIGPI ADLPI and TIGPI GDLHI), but very low similarity with that of hypothetical laccases from *G. luxurians* (KIK69235.1, KIK58304.1, KIK56882.1).

**FIGURE 2:**
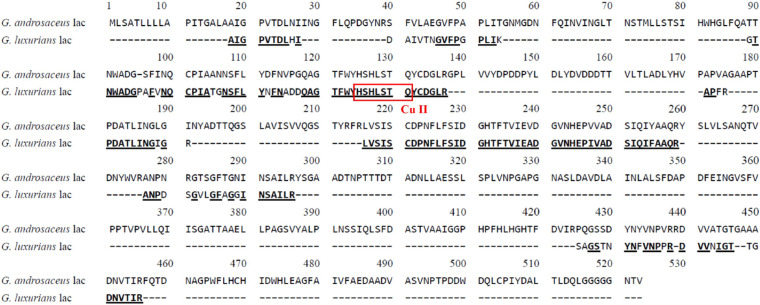
Multiple amino acid sequence alignments of laccase from *G. luxurians* YAASM51591 with hypothetical laccase from *G. androsaceus* (KAE9398736.1).

Six major peptide fragments of GLL by LC-LTQ-Orbitrap-MS were determined as follows: lac1, DAIVT NGVFP GPLIK, lac2, GTNWA DGPAF VNQCP IATGN SFLYN FNADD QAGTF WYHSH LSTQY CDGLR, lac3, APFRP DATLI NGIGR; lac4, LVSIS CDPNF LFSID GHTFT VIEAD GVNHE PIVAD SIQIF AAQR; lac5, ANPDS GVLGF AGGIN SAILR; and lac6, SAGST NYNFV NPPR. A multiple alignment of N-terminal and inner amino acid sequences of GLL with hypothetical laccase from *G. androsaceus* (KAE9398736.1) was constructed using the DNAMAN 8.0 software and is visualized in Figure 2. They demonstrated 90%, 47%, 80%, 58%, 100%, 70%, and 63% similarity with relevant peptide sequence of *G. androsaceus* (KAE9398736.1), respectively. Generally speaking, typical fungal laccases demonstrated four conserved copper-binding motifs, including Cu I (HWHGFFQ), Cu II (HSHLSTQ), Cu III(HPFHLHGH), and Cu IV (HCHIDWHL) (Giardina et al., 2010; Wang et al., 2017). Only Cu II fragments can be found in the LC-MS data. We also tried to clone the core region gene using the degenerate primer combinations based on CuI–CuIV regions. No target sequence similar with laccases was obtained (data not shown). It suggested that the present laccase might be an atypical one.

### Effects of Temperature and pH on Laccase Activity and Stability

**FIGURE 3:**
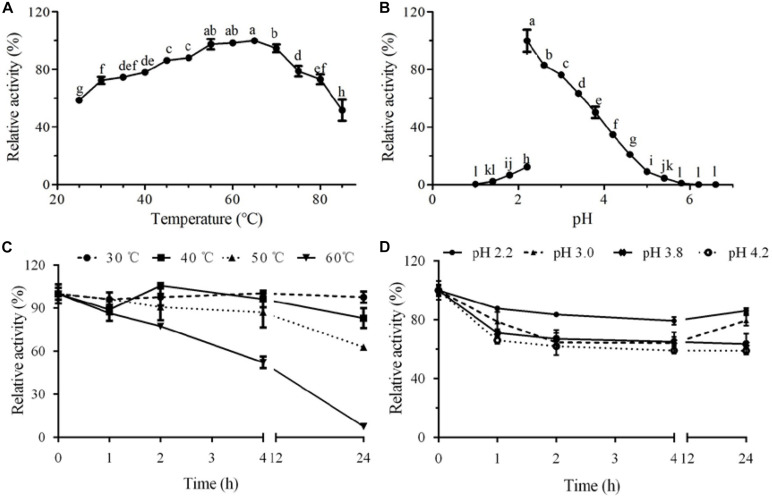
Effect of temperature and pH on activity and stability of the purified laccase GLL. **(A)** optimal temperature; **(B)** optimal pH; **(C)** thermal stability; and **(D)** pH stability. The letters denoted the statistical significance according to the Tukey test at *P* < 0.05.

The purified laccase GLL displayed relatively high activity over a broad temperature range of 25°C –85°C with an optimum temperature range of 55°C–65°C. More than 50% of initial laccase activity remained when it was assayed at 85°C (Figure 3A). The optimal pH value of GLL was determined to 2.2 toward ABTS at 37°C, with about 50% activity loss at pH 3.8 and 95% activity loss at pH 1.8 or 5.4. The oxidizing activity almost vanished when the assay pH was lower than pH 1.4 or higher than pH 5.8 (Figure 3B). The enzyme activity changed dramatically after changing the KCl–HCl buffer system (pH 1.0–2.2) to the citric acid–Na_2_HPO_4_ buffer system (pH 2.2-7.2). It was probably because of the difference of the assayed buffer systems. Similar phenomena exist in other literatures (Xu et al., 2015; Ning et al., 2016). Most fungal laccases are functional toward ABTS at an optimal temperature range of 40°C –70°C and an acidic optimal pH range of 2.0–6.0 (Baldrian, 2006; Viswanath et al., 2014). Laccases from other Marasmiaceae species as *Marasmius* sp., *M. quercophilus* C7, *M. quercophilus* C19, and *M. scorodonius* acted optimally toward ABTS at pH range of 2.6–4.0 and temperature range of 45°C –80°C (Table 3) (Farnet et al., 2002; Farnet et al., 2004; Schuckel et al., 2011; Jeon and Lim, 2017). The purified laccase GLL demonstrates a considerably high temperature optimum range and an acidic pH toward ABTS just like many other fungal laccases reported.

GLL demonstrated considerably high thermostability in the assayed temperature range of 30°C–50°C. After incubation at 50°C for 24 h, about 63% of initial oxidizing activity was maintained. When the assay temperature rose to 60°C, more than half of initial activity (52%) remained after a 4-h incubation (Figure 3C). It is worth mentioning that 86% of initial oxidizing activity maintained after a 24-h incubation at pH 2.2. GLL showed high pH stability in an acidic pH range of 2.2–4.2 with 59%–86% of initial activity remained after a 24-h incubation (Figure 3D). Although the present laccase showed lower oxidation capacity as those laccases from white rot fungi, it manifested better stability toward temperature and pH value. Thermostable laccases have proven to be excellent catalysts in degrading lignin and other aromatic or non-aromatic compounds for their versatile catalytic abilities under industrial conditions and pollution-free by-products (Liu et al., 2020). High optimal temperature and excellent thermostability have bred GLL with better industrial application prospects.

### Effects of Chemical Reagents on Laccase Activity

The effects of metal ions and EDTA toward the purified laccase GLL are shown in Table 4. It is worth mentioning that the oxidizing activity of GLL was significantly enhanced by the presence of common ions as K^+^, Na^+^, and Mg^2+^ at concentrations of 1.25–10 mM with the increasing rate of 22%–73%. The purified laccase was decreased by Ca^2+^, Cd^2+^, Co^2+^, Cu^2+^, Mn^2+^, and EDTA at concentrations of 1.25–10 mM with 40%–90% of the total activity remained. Interestingly, GLL was inhibited by Cu^2+^ ion rather than activation. As copper-containing proteins, many laccases reported can be enhanced by the presence of Cu^2+^ ion, such as *Inonotus baumii* (more than 10-fold increasing at a Cu^2+^ concentration of 1.25–10 mM) (Sun et al., 2014), *Paraconiothyrium variabile* (approximately 50% increasing at a Cu^2+^ concentration of 10 mM) (Forootanfar et al., 2011), *L. edodes* (40% increasing at Cu^2+^ concentration of 10 mM) (Nagai et al., 2002), and *Trametes pubescens* (approximately 10% increasing at Cu^2+^ concentration of 25 mM) (Si et al., 2013). In these laccases, copper can act as the molecular component of the catalytic site or cofactor. Moreover, laccases from *Cerrena unicolor* (approximately 20% decrease) (Wang et al., 2017), *L. naucinus* (approximately 35% decrease) (Ning et al., 2016), and *Fusarium solani* (approximately 30% decrease) (Wu et al., 2010) were inhibited by Cu^2+^ ion at the concentration of 10 or 20 mM. It is similar with the present laccase. The effects of Fe^2+^ and Fe^3+^ ions on laccase activity were not assayed since they can intensively affect the oxidation of ABTS or reduction of ABTS^+^. The oxidation activity of GLL can be efficiently enhanced by light metal as K^+^, Na^+^, and Mg^2+^ instead of Cu^2+^ with low concentrations of 1.25–10 mM, which makes the present laccase more environmentally friendly.

**TABLE 4:** Effect of chemical reagents on the laccase activity of GLL.

Chemical reagent	Relative laccase activity (% of control)			
	1.25 mM	2.5 mM	5 mM	10 mM
K^+^	127.2 ± 4.0 bc	133.0 ± 3.4 b	176.3 ± 4.2 a	125.0 ± 1.5 c
Na^+^	131.0 ± 6.9 ab	139.6 ± 2.1 a	132.3 ± 7.8 ab	123.0 ± 0.7 b
Mg^2+^	122.0 ± 3.5 c	122.9 ± 2.1 c	144.0 ± 1.8 a	135.5 ± 3.8 b
Ca^2+^	87.4 ± 2.4 a	48.4 ± 3.8 b	48.0 ± 0.0 b	40.6 ± 8.4 b
Cd^2+^	85.7 ± 3.2 ab	86.0 ± 14.8 ab	93.3 ± 10.5 a	72.0 ± 3.3 b
Co^2+^	82.4 ± 4.6 a	75.2 ± 8.7 ab	63.6 ± 6.2 b	63.1 ± 6.2 b
Cu^2+^	67.3 ± 4.9 ab	72.7 ± 1.5 a	61.5 ± 2.2 b	60.4 ± 2.7 b
Mn^2+^	89.5 ± 4.2 a	89.5 ± 2.5 a	89.7 ± 7.2 a	91.8 ± 1.2 a
EDTA	84.9 ± 4.9 b	65.9 ± 7.8 b	70.2 ± 7.8 a	74.5 ± 4.3 c

*Laccase activity in the absence of metal ions was regarded as 100%. Values represent mean ± standard deviation (*n* = 3). The letters denoted the statistical significance according to the Tukey test at *p* < 0.05.*

### Determination of Enzyme Kinetics

Kinetic analysis was carried out with ABTS as the substrate at pH 2.2 and 37°C. *K*_*m*_, *V*_*max*,_ *k*_*cat*_, and *k*_*cat*_/*K*_*m*_ of the purified laccase GLL were calculated to be 539 μM, 3.1 μmol/min, 75.4 s^–1^, and 140 mM^–1^⋅s^–1^, respectively. *K*_*m*_ and *k*_*cat*_/*K*_*m*_ are enzymatic kinetic parameters which can reflect the affinity toward substrates and catalytic efficiency. Fungal laccases demonstrate a very broad *K*_*m*_ value range toward ABTS of about 4–2500 μM (Baldrian, 2006; Viswanath et al., 2014). GLL possessed a considerably relatively low *K*_*m*_ value, suggesting that it displayed high affinity toward ABTS. Comparisons of enzymatic kinetic parameters of GLL and laccase from relative species of genus *Marasmius* are listed in Table 3. Laccase from *Marasmius* sp., *Marasmius* sp. BBKAV79, *M. quercophilus* C7, *M. quercophilus* C19, and *M. scorodonius* displayed *K*_*m*_ values toward ABTS of 3.9, 3030, 7700, 113, and 27 μM, respectively (Farnet et al., 2002, 2004; Schuckel et al., 2011; Jeon and Lim, 2017). GLL possessed higher affinity toward ABTS than the laccase from *Marasmius* sp. BBKAV79 and *M. quercophilus* C7, but lower than the other three. Moreover, the catalytic efficiency of GLL, based on the *k*_*cat*_/*K*_*m*_ ratio, was lower than those of *Marasmius* sp. and *M. scorodonius*.

### Optimal Mediators and Reaction Conditions for Dye Decolorization

**FIGURE 4:**
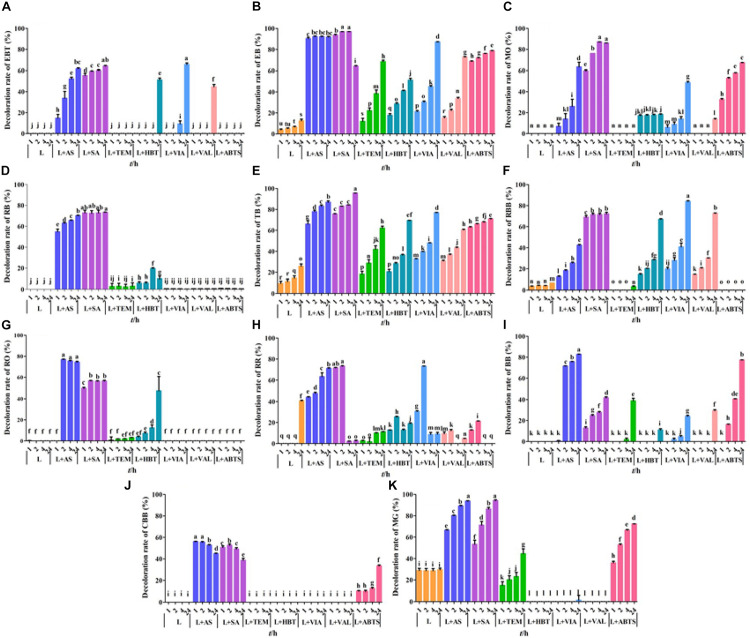
Dye decolorization by LMS. **(A)** eriochrome black T (EBT); **(B)** Evans blue (EB); **(C)** methyl orange (MO); **(D)** reactive black (RB); **(E)** trypan blue (TB); **(F)** reactive brilliant blue (RBB); **(G)** reactive orange (RO); **(H)** reactive red (RR); **(I)** bromophenol blue (BB); **(J)** Coomassie brilliant blue G250 (CBB); **(K)** malachite green (MG). L, free laccase; AS, acetosyringone, SA, syringaldehyde; TEMPO, 2,2,6,6-tetramethylpiperidine 1-oxyl; HBT, 1-hydroxybenzotriazole; VIA, violuric acid; VAL, vanillin; ABTS: 2,2′-azino-bis (3-ethylbenzothiazoline-6-sulfonic acid). The letters denoted the statistical significance according to the Tukey test at *P* < 0.05.

Dye decolorization capacity of GLL and seven mediator systems were assayed toward 14 dyes (Figure 4). The free laccase GLL from the litter-decomposing fungus *G. luxurians* displayed decolorization ability toward assayed dyes with the maximum decolorization rate of 40.5% toward RR after a 24-h incubation, followed by MG (29.5%) and TB (25.7%). It can hardly degrade the others. Zheng et al. (2017) reported that a laccase from white rot fungus *Trametes orientalis* demonstrated considerably high decolorization ability (over 50%) toward acid black 172, Congo red, neutral red, etc. Compared with many efficient dye decomposers from white rot fungi (Erkurt et al., 2007; Kumar et al., 2012; Arregui et al., 2019), the free laccase GLL displayed relatively low decolorization ability. On the other hand, it was more thermostable and pH stable. Therefore, we added small-molecule redox mediators to improve the decolorization efficiency and further potential application of the purified laccase. Among the seven assayed mediators, AS and SA were determined to be the optimal mediators for dye decolorization. They can significantly improve the decolorization ability of GLL toward azo and triphenylmethane dyes. After a 24-h incubation, LMS of GLL-AS or GLL-SA possessed a considerably high decolorization rate of about 60%–90% toward 11 of the 14 assay dyes. GLL-AS and GLL-SA displayed very efficient degradation capability toward EB with the decolorization rates 90.4% and 93.6% after a 1-h incubation, respectively. Moreover, the decolorization ability of the laccase toward reactive dyes of AR, RTB, and RY was still very low (0%–20%) even in the presence of a mediator (data not shown).

**FIGURE 5:**
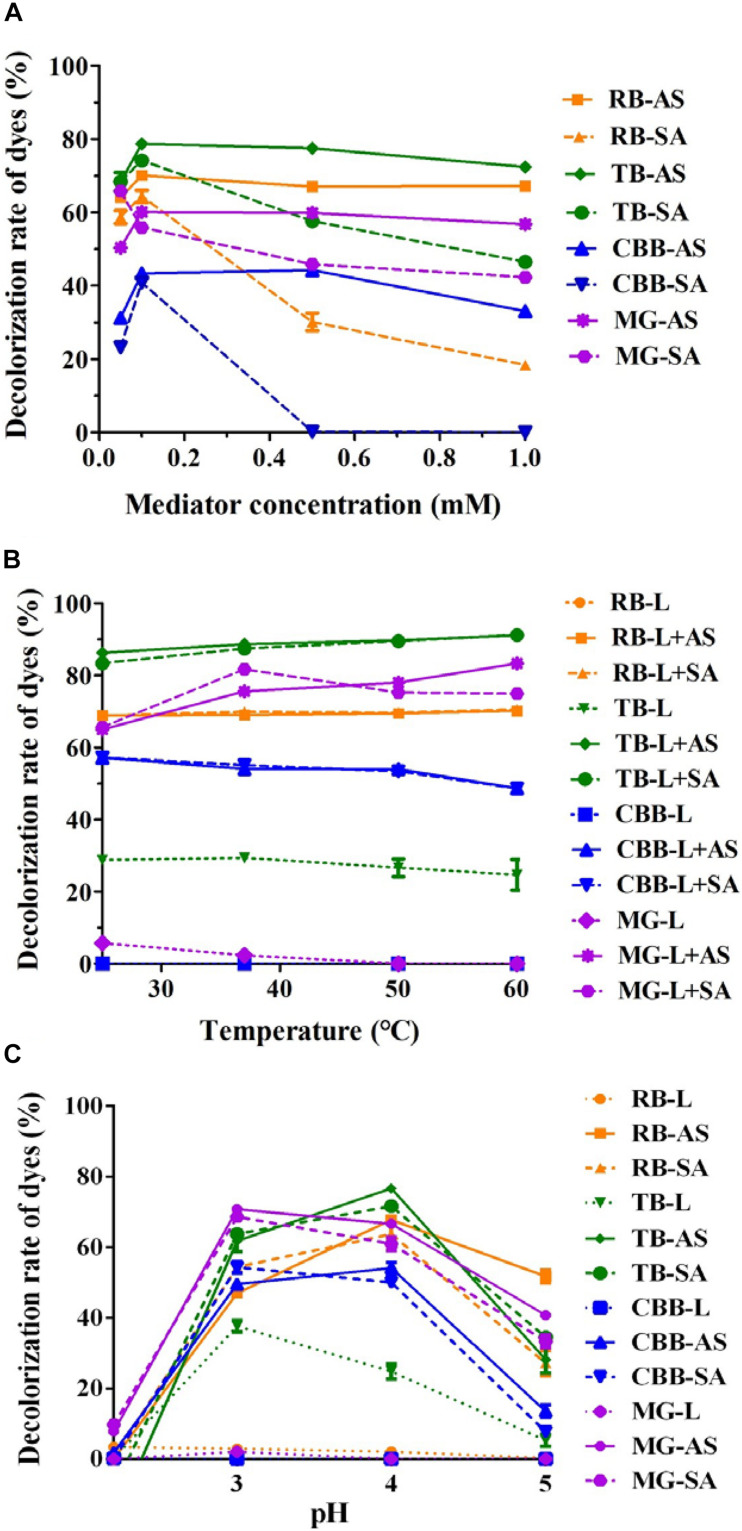
Optimal LMS reaction conditions for dye decolorization. **(A)** Mediator concentration; **(B)** temperature; **(C)** pH.

**FIGURE 6:**
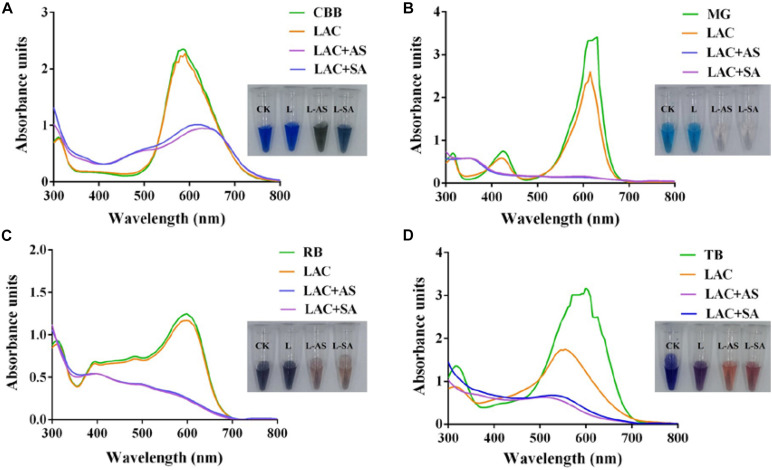
Full-wavelength scanning (300–800 nm) of reaction products of LMS for dye decolorization. **(A)** Coomassie brilliant blue G250 (CBB); **(B)** malachite green (MG); **(C)** reactive black (RB); **(D)** trypan blue (TB).

To further improve the decolorization efficiency, the optimal reaction conditions of GLL-AS and GLL-SA toward four dyes of RB, CBB, MG, and TB were performed. The optimal mediator concentration, temperature, and pH value were determined to be 0.1 mM, 25°C–60°C, and pH 4.0, respectively, with decolorization rates of about 50%–90% toward the assayed dyes after a 4-h incubation (Figure 5). Ultimately, decolorization characteristics of free laccase and LMS toward the four assayed dyes were determined at the optimal conditions including a mediator concentration of 0.1 mM, 60°C (also the optimal temperature of GLL), and pH 4.0. After a 4-h incubation, the decolorization rate of LMS (GLL-AS and GLL-SA) almost achieved the highest level of about 50%–90%, which was tremendously higher than that of free laccase. Full-wavelength scanning of reaction products of LMS for the four assayed dyes is visualized in Figure 6. The maximum absorption peak of MG (at about 615 nm) and TB (at about 600 nm) almost vanished. Meanwhile, similar results were obtained from CBB and RB group with absorption decrease less than half.

Recently, LMS has been widely studied since its great prospects in environmental pollution control, textile industry, food industry, modification of lignocellulose materials, etc. (Kudanga et al., 2011; Jeon and Chang, 2013; Ozer et al., 2019). Laccase activity of *T. hirsuta* Bm-2 with ABTS showed the highest level of dye decolorization (97%) compared with laccase alone (12%) (Ancona-Escalante et al., 2018). VA, AS, and TEMPO were the optimal mediators of the laccase from *Trametes* sp. F1635 for dye decolorization (Wang et al., 2018). The decolorization activity of laccase from *Myceliophthora thermophila* can be enhanced by the redox mediators as HBT, SA, catechol, and N-hydroxyphthalimide (HPT) (Salami et al., 2018). The present laccase possesses considerable high dye decolorization activity at the presence of AS and SA. The highest decolorization rate of GLL-AS and GLL-SA toward MG was determined to be 94.6% and 89.2%, respectively, after a 4-h incubation, while that of GLL was only 6.6%. Both AS and SA are occurring derivatives of syringic acid which is one of the abundant phenolic compounds in lignin and has an effective free radical scavenger and alleviates the oxidative stress markers (Srinivasulu et al., 2018). They may participate in free radical transfer during the laccase-induced dye decolorization (Morozova et al., 2007; Canas and Camarero, 2010).

## Conclusion

In summary, a novel laccase GLL was purified from *G. luxurians* with commendable thermostability and pH stability, which is the first purified laccase from genus *Gymnopus*. It possessed different enzymatic characteristics from other laccases of family Marasmiaceae. Furthermore, GLL with some small-molecule redox mediators as AS and SA demonstrated efficient decolorization capability toward synthetic dyes. It is suggested that the purified laccase demonstrates potential application in textile and environmental industries.

## Data Availability Statement

The original contributions presented in the study are included in the article/supplementary material, further inquiries can be directed to the corresponding author/s.

## Author Contributions

YS, Z-LL, B-YH, Q-JC, A-ZY, Q-YW, X-FL, and J-YZ designed and performed the experiments and analyzed the data. Y-CZ provided the strain and conceived and designed the experiments. G-QZ conceived and designed the experiments and wrote the original manuscript. All authors contributed to the article and approved the submitted version.

## Conflict of Interest

The authors declare that the research was conducted in the absence of any commercial or financial relationships that could be construed as a potential conflict of interest.

## Publisher’s Note

All claims expressed in this article are solely those of the authors and do not necessarily represent those of their affiliated organizations, or those of the publisher, the editors and the reviewers. Any product that may be evaluated in this article, or claim that may be made by its manufacturer, is not guaranteed or endorsed by the publisher.

## Footnotes

1
https://mycocosm.jgi.doe.gov/Gyman1

2
https://mycocosm.jgi.doe.gov/Gymear1

3
https://mycocosm.jgi.doe.gov/Gymlu1
